# Death after sauna—case report of a heat stroke associated subarachnoid hemorrhage

**DOI:** 10.3389/fcvm.2024.1476962

**Published:** 2024-11-26

**Authors:** Anna Maria Nothnagel, Stefan Schwartz, Igor Abramovich, Thomas Schneider, Stefan Angermair, Sascha Treskatsch

**Affiliations:** ^1^ Freie Universität Berlin, Humboldt Universität zu Berlin, and Berlin Institute of Health, Department of Anesthesiology and Intensive Care Medicine, Campus Benjamin Franklin, Charité - Universitätsmedizin Berlin, Berlin, Germany; ^2^ Freie Universität Berlin, Humboldt Universität zu Berlin, and Berlin Institute of Health, Department of Haematology, Oncology and Tumour Immunology, Campus Benjamin Franklin, Charité - Universitätsmedizin Berlin, Berlin, Germany; ^3^ Freie Universität Berlin, Humboldt Universität zu Berlin, and Berlin Institute of Health, Department of Anesthesiology an Intensive Care Medicine (CCM/CVK), Charité - Universitätsmedizin Berlin, Berlin, Germany; ^4^ Freie Universität Berlin, Humboldt Universität zu Berlin, and Berlin Institute of Health, Department for Gastroenterology, Infectiology and Rheumatology, Campus Benjamin Franklin, Charité - Universitätsmedizin Berlin, Berlin, Germany

**Keywords:** heat stroke, disseminated intravascular coagulation, sauna, subarachnoid hemorrhage, case report

## Abstract

Heat exposure could cause organ injuries leading to multi-organ failure. This case report describes a cardiopulmonary healthy 61-year-old woman who was admitted to our university hospital in an unconscious state after spending more than 30 min in an 80°C sauna. Initial radiological imaging revealed no pathological findings. Laboratory results revealed a rapidly progressing disseminated intravascular coagulation (“DIC”) and a clinically asymptomatic COVID-19 infection. The patient died 38 h after admission due to a second-stage subarachnoid hemorrhage associated with progressive DIC. This report emphasizes the importance of awareness and strategies to mitigate deadly consequences of heat exposure.

## Introduction

Sauna is known as a wellbeing or health supporting treatment, which is widespread all over the world. Health benefits have been reported for hundreds of years. Regular sauna visits can improve vascular function in patients with known heart disease and/or risk factors, treat muscular tension and could support mental health treatment (1). However, external heat exposure comes along with the formal danger of fluid loss, fainting and finally heat stroke.

Heat stroke is a potentially life-threatening condition caused by excessive heat exposure and poor thermoregulation. Heat stroke is a common medical problem in especially hot and humid climate regions all over the world. However, the same medical condition could appear while visiting a sauna for far too long. Body temperature may rise sharply due to the high ambient temperatures, potentially leading to a dysfunction of the central nervous system and a systemic inflammatory response, which can lead to multi-organ failure (2).

In addition, hyperthermia is often accompanied by coagulation disorders (3–5). There exist case reports describing harmful effects of sauna heat exposure (6). Because of the potentially lethal effects, it is important to raise awareness of risk factors, symptoms and treatment options of heat stroke associated with sauna, which will be presented in this case report.

Written consent to publication for this case report was confirmed by the hospital's internal university medical treatment contract and from the patient's relative.

## Description

A cardiopulmonary healthy 61-year-old female patient was found lying unconscious by her husband in the domestic 80°C sauna after a stay of at least 30 min. Due to uncertain vital signs and persistent myocloni, lay resuscitation was started.

Medical history revealed a leiomyosarcoma of the sigmoid and colon, which had been completely resected several years ago. The woman did not take any medication or noxious substances, had no allergies, and led an active, sporty lifestyle. In the week before the event, the patient was on a skiing holiday with her husband and friends. Due to a COVID-19 infection detected in the immediate vicinity afterwards, daily self-tests were carried out, which had always been negative until then. The patient had received a total of 3 vaccinations by the time of hospital admission. In addition, the patient went regularly (several times a week) to the 80°C sauna; according to her husband, the normal duration of stay was always about 20–30 min.

On arrival of the ambulance service, the patient presented with a Glasgow Coma Scale 3/15, periodic contractions of the arms, a tachycardic sinus rhythm, hypotension with a mean arterial pressure of 50mmHg and a SpO2 of 81%. The initial tympanic temperature measured on-site was 37.7°C only. Endotracheal intubation was performed without delay, mechanical ventilation was initiated, and the patient was transported to the emergency department of our university hospital.

Neurological examination in the emergency department confirmed myoclonus with periodic contractions of both arms without clear synergism. There was no evidence of tongue bits, pupils were conjugated and narrow bilaterally, but a pupillary response could not be elicited with certainty. The Babinski reflex was negative on both sides; the patient was normoglycemic. Burn marks were apparent on both knees, there were no others trauma signs found.

Blood pressure on admission in the emergency room was 100/60 mmHg without vasopressor support in conjunction with a tachycardic sinus rhythm of 130/bpm without excitation-recovery disturbances.

In synopsis, a seizure due to hyperthermia coupled with the COVID-19 infection and weakness was considered as the most possible cause for unconsciousness at this moment. Therefore and because of insufficient analgo-sedation, further intravenous medication with 5 mg midazolam and additionally 1 g levetiracetam as well as a propofol infusion (2 mg/kg/min) were administered in the emergency room. This allowed myocloni to be interrupted and a consecutive tube tolerance be be achieved.

Focused Assessment with Sonography for Trauma ultrasound showed no pathology. Focused transthoracic echocardiography excluded regional wall motion abnormalities, right ventricular dysfunction, and pericardial effusion. Cranial and body computed tomography angiography (“CT-A”) revealed no pathologies suggestive of intracranial hemorrhage, edema, trauma, tumor, infarction, cerebral vascular occlusion or vascular malformation. CT body scan also ruled out pulmonary artery embolism, pneumothorax and pleural effusions. Likewise as in the mentioned ultrasound examination, there was no evidence of a right heart dysfunction nor pericardial effusion. Only a fine-spotted compaction was detected in the dorsobasal lower lobe on the right side.

Laboratory results excluded any intoxication, however, the COVID-19 rapid test with polymerase chain reaction (“PCR”) was highly positive.

**Figure 1:**
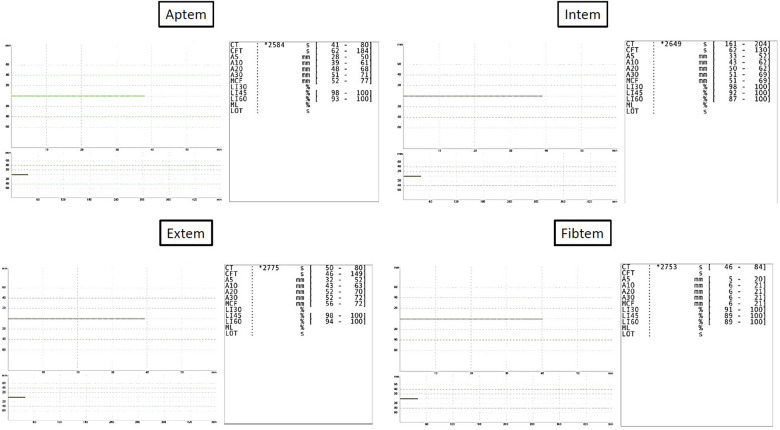
Results of rotational thromboelastometry.

After initial treatment in the emergecy room, the patient was transferred to our intensive care unit under controlled ventilation for further treatment. No administration of vasopressors was necessary to maintain adequate mean arterial pressure. Notably, coagulation parameters were conspicuous as shown in Table 1. Rotational thromboelastometry (“ROTEM™”) was thus performed for extended coagulation diagnostics immediately after admission on the Intensive Care Unit. ROTEM™ results indicated an almost complete inapparency of all measured coagulation factors in all derivations (Figure 1). To achieve a rapid improvement in coagulation, substitution of 2,400 IU of Prothrombin concentrate, 2 platelet concentrates, 8 g of fibrinogen, 2,150 IU of factor XIII and 1 g of tranexamic acid as a bolus and an additional 1 g over 8 h for highly suspected disseminated intravascular coagulation was performed. The first arterial blood gas analysis showed a mild mixed respiratory and metabolic acidosis, as shown in Table 2. Temperature was continuously measured via an inserted urinary catheter after ICU admission. The patient was consistently normoterm (35.8° to a maximum of 37.7°C), so that no specific therapy was needed throughout ICU treatment. Analgo-sedation was stopped in order to achieve a rapid neurological assessment. Unfortunately, the patient did not provide an adequate wake-up response.

**Table 1:** Laboratory results at inpatient admission.

Name	Unit	Value (reference range)	Name	Unit	Value (reference range)
Troponin	ng/L	242 (<14)	Creatinin	mg/dL	1.48 (0.5–0.9)
Ferritin	µg/L	4,520 (13–150)	Albumin	g/dL	39 (35–52)
GPT/ALT	U/L	21 (<31)	INR		1.38 (0.9–1.25)
GOT/AST	U/L	69 (<35)	aPTT	Sec.	53 (25–38)
ALP	U/L	52 (35–105)	PCT	µg/L	0.05 (<0.5)
CK	U/L	191 (<167)	TSH	mU/L	2.02 (0.27–4.2)
Lipase	U/L	445 (13–60)	Leukocytes	/nL	5.65 (3.9–10,5)
CRP	mg/L	5.4 (<5)	Erythrocytes	/nL	3.6 (3.9–5.2)
TSH	mU/L	2.02 (0.27–4.2)	Hb	g/dL	11.1 (12–15.6)

Values in the Normal Range according to the Reference of the Laboratory “Labor Berlin”.

aPPT, activated partial thromboplastin time; ALP, alkaline phosphatase; CK, Creatine kinase; CRP, C-reactive protein; GPT/ALT, glutamate pyruvate transaminase/alanine aminotransferase; GOT/AST, glutamate oxaloacetate transaminase/aspartate aminotransferase (glutamate oxaloacetate transaminase/aspartate aminotransferase); HB, haemoglobin; HK, haematocrit; INR, International Normalised Ratio; PCT, Procalcitonin; TSH, thyroid stimulating hormone.

**Table 2:** Laboratory results of the first arterial blood gas analysis after admission on the ICU.

FiO2%	0.5
PH	7.262
pO2 mmHg	216
PCO2 mmHg	47.0
sO2%	98.7
HCO3 mmol/L	20.5
SBE mmol/L	−5.4
Hb g/dL	14.6
Hkt%	44.9
MetHb%	0.8
CO_Hb%	0.0
Sodium mmol/L	145
Potassium mmol/L	4.5
Lactate mg/dL	17
Glucose mg/dL	154

Differential diagnosis of an epileptic seizure was ruled out by a continuous EEG recording. Further blood analysis ruled out intoxication with alcohol, opiates, barbiturates, benzodiazepines, amphetamines, cannabinoids, methadone, lithium, paracetamol and salicylic acid. In the following also other rare pathophysiological causes were excluded by laboratory testing, e.g., Hantavirus infection, Campylobacter and Rickettsia infection, Cytomegaly virus, herpes, virus-induced hepatitis and haemapoetic disorders. Finally, normal thyroid hormones ruled out thyroid disorders.

**Figure 2:**
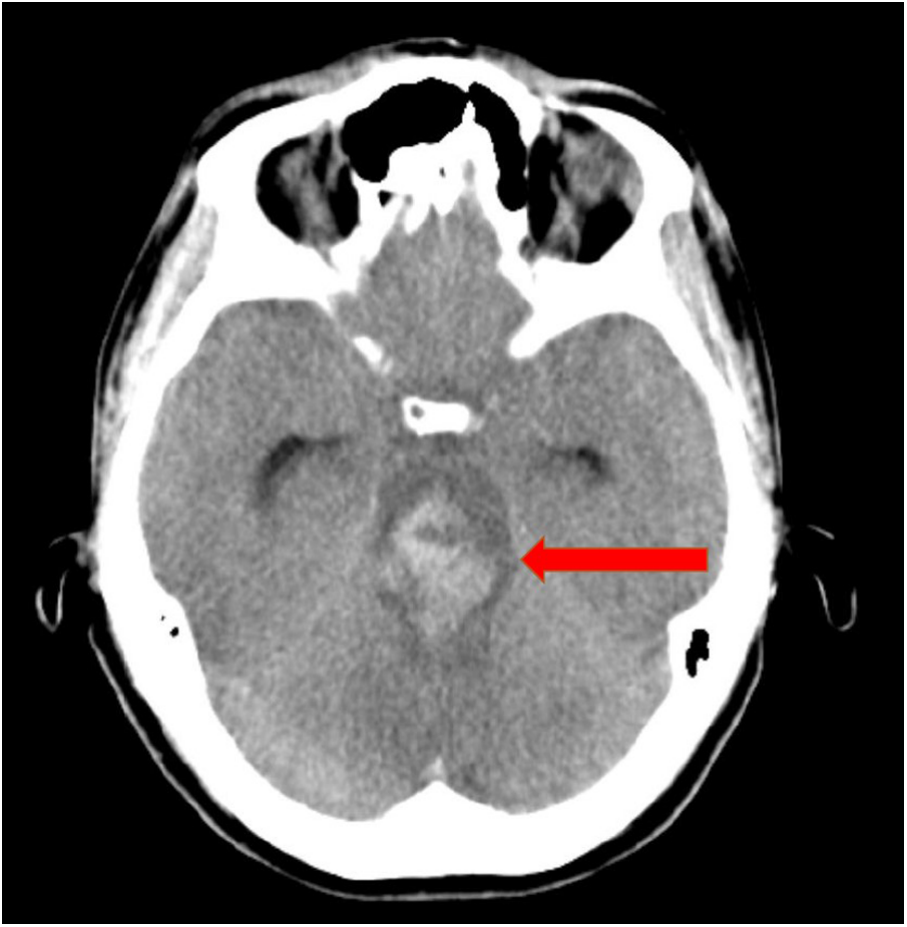
CT angiography shows extensive subarachnoid hemorrhage in the area of the mesencephalon and pons.

A lumbar puncture was not conducted due to the coagulation disorder. The next day during the 2-hourly check-up, both of the patient's pupils suddenly appeared wide and no longer reactive to light. By interdisciplinary consensus, it was decided to immediately repeat CT-A. It showed extensive subarachnoid hemorrhage with generalized abolition of medullary cortical contrast and signs of incipient herniation (Figure 2). Due to the imaging findings with pronounced brain stem hemorrhage, the patient's prognosis was assessed as inauspicious within a team of neurologists, neurosurgeons, haemato-oncologists and intensive care physicians. Intensive care measures were thus terminated within a shared decision process including the patient´s familiy. The patient died 38 h after ICU admission after palliative termination of mechanical ventilation accompanying adequate symptom control.

Because of the history of leiomyosarcoma and the rapid changes in coagulation, a follow-up post-mortem bone marrow biopsy was performed. This showed signs of most likely toxin-induced dysplasia without signs of (acute) myelodysplastic syndrome. As the cause of death was still unclear, a forensic medical examination was ordered.

Here, the space-occupying hemorrhage in the brain stem was confirmed as the leading cause of death. The anatomical-pathological report also showed further hemorrhages, both inside and outside the pericardium, as well as ubiquitous petechiae and small hematoma of the skin. In addition, as a sign of thermal exposure, there was widespread redness over both knees and partial blister-like detachment of the skin on the right lower leg. No relevant pathogens were found in the blood cultures or smears.

## Discussion

There exist several case reports of deaths after sauna visits, which are mainly combined with vascular diseases or intoxications (6). But heat exposure in general is a risk factor contributing to cardiovascular disease (7). Disturbances of consciousness up to coma, inflammatory response, coagulation disorder as disseminated intravascular coagulation, and consecutive organ failure is typical for a severe heat stroke. Despite adequate treatment, the risk of mortality is up to 30%, and thousands of people die every year of this potentially fatal disease (4, 8). In particular, many patients die from a delayed coagulation disorder, which cause spontaneous bleeding on mucous membranes, such as gastrointestinal hemorrhages (8). However, fatal spontaneous cerebral hemorrhage may also occur as in the here presented case (2, 5).

Our patient's initially measured temperature was only 37.7°C, but the relationship between body temperature and heat stroke has not been clearly defined or classified (9). It appears that although the temperature measurement excluded severe heat stroke pro forma, and the COVID-19 infection was asymptomatic, the combination of both conditions may have catalyzed the fatal course. The husband noticed that our patient did not feel very well this evening, most likely due to the viral infection. Probably she fell asleep in the sauna, while relaxing and being tired at the end of the day.

This in turn would indicate that the sauna visit might have been far longer than 30 min, increasing the risk of dehydration and thus heat stroke.

The patient had no symptomatic COVID-19 infection in terms of pulmonary dysfunction and no evidence of typical infiltrates on computed tomography. Nevertheless, IL-6 of 9,544 ng/L and ferritin of 4,520 µg/L were both massively elevated, as found in most severe COVID-19 infections. As a result, it must probably be assumed that the here presented course of disease has been negatively affected by COVID-19.

Coronavirus disease causes among others severe thrombosis and DIC (10). Although DIC associated with COVID-19 infection usually presents differently (11), here it may be postulated that the infection in combination with heat exposure contributed to the more rapid development of the disease. Early use of ROTEM™ may help predicting DIC associated with heatstroke (12).

Heat stroke is still a fatal disease, especially if it is not recognized and quickly treated. Usual therapy aiming at rapid recovery of normothermia and intensive medical treatment of organ dysfunctions such as cerebral edema, acute kidney damage and coagulation failure as a consequence of acute liver failure, must be carried out as soon as possible after a hyperthermia-induced disease has been detected (13).

Our case report highlights that isolated temperature measurements - especially at a later time in relation to the onset of the emergency and in the presence of normothermia - are not sufficient to rule out thermally induced organ damage. In addition, there seems to exist a knowledge gap in the treatment of heatstroke-related complications as genetically determined individual vulnerability to heat stress is unknown. This emphasizes the need for further research to better understand the pathophysiology of heat illness, heat stroke and associated multi-organfailure with its often lethal outcome.

## Data Availability

The raw data supporting the conclusions of this article will be made available by the authors, without undue reservation.
